# Computer-aided drug design approaches applied to screen natural product’s structural analogs targeting arginase in Leishmania spp

**DOI:** 10.12688/f1000research.129943.3

**Published:** 2023-07-13

**Authors:** Haruna Luz Barazorda-Ccahuana, Luis Daniel Goyzueta-Mamani, Mayron Antonio Candia Puma, Camila Simões de Freitas, Grasiele de Sousa Vieria Tavares, Daniela Pagliara Lage, Eduardo Antonio Ferraz Coelho, Miguel Angel Chávez-Fumagalli

**Affiliations:** 1 Computational Biology and Chemistry Research Group, Vicerrectorado de Investigación, Universidad Catolica de Santa Maria de Arequipa, Arequipa, Peru; 2 Sustainable Innovative Biomaterials Department, Le Qara Research Center, Arequipa, Peru; 3 Universidad Católica de Santa María, Facultad de Ciencias Farmacéuticas, Bioquímicas y Biotecnológicas, Arequipa, Peru; 4 Universidade Federal de Minas Gerais, Programa de Pós-Graduação em Ciências da Saúde: Infectologia e Medicina Tropical, Faculdade de Medicina, Belo Horizonte, Minas Gerais, Brazil; 5 Universidade Federal de Minas Gerais, Departamento de Patologia Clínica, COLTEC, Belo Horizonte, Minas Gerais, Brazil

**Keywords:** leishmaniasis, Leishmania arginase, computer-aided drug design, molecular dynamics simulation, antiprotozoal agents; drug discovery

## Abstract

**Introduction:** Leishmaniasis is a disease with high mortality rates and approximately 1.5 million new cases each year. Despite the new approaches and advances to fight the disease, there are no effective therapies.

**Methods:** Hence, this study aims to screen for natural products' structural analogs as new drug candidates against leishmaniasis. We applied Computer-aided drug design (CADD) approaches, such as virtual screening, molecular docking, molecular dynamics simulation, molecular mechanics–generalized Born surface area (MM–GBSA) binding free estimation, and free energy perturbation (FEP) aiming to select structural analogs from natural products that have shown anti-leishmanial and anti-arginase activities and that could bind selectively against the *Leishmania* arginase enzyme.

**Results:** The compounds 2H-1-benzopyran, 3,4-dihydro-2-(2-methylphenyl)-(9CI), echioidinin, and malvidin showed good results against arginase targets from three parasite species and negative results for potential toxicities. The echioidinin and malvidin ligands generated interactions in the active center at pH 2.0 conditions by MM-GBSA and FEP methods.

**Conclusions:** This work suggests the potential anti-leishmanial activity of the compounds and thus can be further *in vitro* and *in vivo* experimentally validated.

## Introduction

Leishmaniasis is an ancient disease that has been described in archaic ceramics, statues, and writings, and in molecular findings from mummified human bodies and archaeological material. ^1^ The disease causes high morbidity and mortality worldwide, where about one billion people are at risk of infection across 98 countries, with over 1.5 million new cases and 20,000-40,000 deaths reported each year. ^2^^,^^3^ The increase in leishmaniasis incidence and prevalence is mainly attributed to several risk factors that are man-propelled, ^4^ whereas, in many regions, the transmission pattern shows expansion, with new territories affected by the disease. ^5^^,^^6^ Also, leishmaniasis has gained greater importance in HIV-infected patients as an opportunistic infection in areas where both pathogens are endemic. ^7^ Leishmaniasis is caused by the protozoan parasites of the genus *Leishmania* (Kinetoplastida: *Trypanosomatidae*), which has a digenetic life cycle that alternates between the midgut of sandflies and the phagolysosomes of mammalian macrophages. ^8^ When exposed to extreme environmental changes, such as low pH, the parasites respond to the acidification of their environment by changing the pattern of expression of several proteins. ^9^^,^^10^ About 21 parasite species can infect mammals and many of them cause human disease ^11^ and the clinical manifestations depend on both the parasite species and the hosts’ immune response, ^12^ varying from a chronic, slow-to-heal disease known as tegumentary leishmaniasis (TL), to a potentially fatal form of the disease, namely, visceral leishmaniasis (VL), in which parasites disseminate to internal organs, such as the liver, spleen, and bone marrow. ^13^

Despite significant progress, the development of a human vaccine remains hampered by significant gaps in the development pipeline ^14^; and the treatment against disease has used drugs that cause side effects in the patients, such as myalgia, arthralgia, anorexia, fever, and urticaria, as well as toxicity in the liver, kidneys, and spleen. ^15^ Therefore, the necessity for cost-effective treatment which promotes the cure completely, with few side effects, low relapse rates, high effectiveness, and a reduction of toxicity remains. ^16^ The number of drugs derived from natural products (NPs) present in the total amount of drug launchings in the market over four decades represents a significant source of new pharmacological entities, ^17^ while a series of secondary plant-purified products has already been described with leishmanicidal potential. ^18^^–^^21^ Likewise, computer-aided drug design (CADD) can be defined as computational approaches that are used to discover, develop, and analyze drug and active molecules with similar biochemical properties, ^22^ and this has become crucial for screening of potential metabolite databases from natural sources that can be repurposed against diseases for faster, safer, and cheaper drug development. ^23^^,^^24^ The strategy of target-based drug discovery is used extensively by the pharmaceutical industry and has been applied to leishmaniasis. ^25^^,^^26^ However, *in silico* methods to identify new potential drugs to be applied against leishmaniasis present limitations, such as the dependency on the quality, accuracy, and completeness of the information present in databases. ^27^ The arginase (ARG) enzyme has recently obtained considerable attention since new studies have highlighted it as a potential therapeutic target in leishmaniasis. ^28^ ARG is the first enzyme of the polyamine pathway and catalyzes the conversion of L-arginine to L-ornithine and urea, down-regulating the polyamine pathway, affecting the parasite growth and infectivity. ^29^ The inhibition results in a lack of protection against reactive oxygen species (ROS), which damages *Leishmania*’s genetic material and ultimately leads it to die by apoptosis. ^30^ As a result, various NPs have demonstrated anti-arginase action, ^31^^,^^32^ and the majority of these NPs have also demonstrated a strong affinity against human ARG. ^33^ In the current study, we used CADD techniques, such as virtual screening, molecular docking, and molecular dynamics simulations, to identify structural analogs of NPs that have demonstrated anti-leishmanial and anti-ARG activities and that may bind specifically to the *Leishmania* ARG. Our goal was to identify a promising compound candidate that could be used in the treatment of leishmaniasis.

## Methods

### Data collection

The search for natural products with anti-leishmanial and anti-ARG activities was performed at the Nuclei of Bioassays, Ecophysiology, and Biosynthesis of Natural Products Database (NuBBEDB) online web server (version 2017) ( http://nubbe.iq.unesp.br/portal/nubbe-search.html, accessed on 23 January 2022), which contains the information of more than 2,000 natural products and derivatives ^34^; while the “anti-leishmanial property” was selected in the biological properties segment of the web server. The bibliographic data extraction, regarding the compounds found in NuBBEDB, was performed from the National Center for Biotechnology Information (NCBI) databases ( https://www.ncbi.nlm.nih.gov/pubmed/, accessed on 07 February 2022); and the simplified molecular-input line-entry system (SMILES) was searched and retrieved from PubChem server ( https://pubchem.ncbi.nlm.nih.gov/, accessed on 10 February 2022). ^35^ Likewise, the physicochemical properties: total molecular weight (MW), octanol/water partition coefficient (iLOGP), number of H-bond acceptors (HBAs), number of H-bond donors (HBDs), and the topological polar surface area (TPSA), for each compound were calculated within the Osiris DataWarrior v5.2.01 software ^36^; and, the rotatable bonds (RB); number of heavy atoms (NHA); and synthetic accessibility (SynAcce) were calculated within SwissADME server ( http://www.swissadme.ch/index.php, accessed on 15 February 2022). ^37^

### Structural analogs search and virtual screening

The SMILES from the compounds were used for high throughput screening to investigate structural analogs by the SwissSimilarity server ( http://www.swisssimilarity.ch/index.php, accessed on 01 March 2022) ^38^; whereas the commercial class of compounds was selected and the Zinc-drug like compound library, which comprises 9’205’113 molecules, with the combined screening method, was chosen for the high throughput screening to achieve the best structural analogs. The zinc-drug like compound library selection allowed the screening of compounds in the subsequent commercially available chemical libraries: Enamine, ChemBridge, Maybridge, Asinex, AsisChem, Otava, SPECS, TimTec, Vitas, Life Chemicals, ChemDiv, and Innovapharm. ^39^ Threshold values for positivity were selected by default parameters. Also, the FASTA sequences of the ARG sequences from *L. infantum* (A4IB49), *L. mexicana* (Q6TUJ5), *L. braziliensis* (A4HMH0), and *Homo sapiens* (P05089) were retrieved from UniProt database ( http://www.uniprot.org/, accessed on 03 March 2022) (RRID:SCR_002380), and subjected to automated modeling in SWISS-MODEL ^40^ (RRID:SCR_018123), whereas the best model was selected based on the GQME and QMEAN4 scores.

Furthermore, the compounds were imported into Open Babel (RRID:SCR_014920) within the Python Prescription Virtual Screening Tool ^41^ and subjected to energy minimization. PyRx (RRID:SCR_018548) performs structure-based virtual screening applying molecular docking simulations using the AutoDock Vina tool ^42^ (RRID:SC_011958), whereas the drug targets were uploaded as macromolecules. For the analysis, the search space encompassed the whole of the modeled 3D models, and the molecular docking simulation was then run at an exhaustiveness of 8 and set to only output the lowest energy pose. The Osiris Data Warrior software was employed to calculate the potential tumorigenic, mutagenic, and reproductive effects, and irritant action of selected compounds predicted by comparison with a precompiled fragment library derived from the Registry of Toxic Effects of Chemical Substances (RTECS) database. ^36^

### Molecular dynamics simulation

Ligands preparation was based on the results from the virtual screening analysis; while the geometry optimization of these compounds was made in the Avogadro v. 1.2.0 program ^43^ (RRID:SCR_015983) and the ACPYPE (AnteChamber PYthon Parser interfacE) ^44^ server was employed to generate the topologies and parameters for molecular dynamics (MD) simulation. We determined the 3D structural conformation of *L. infantum* ARG by homology modeling with *L. mexicana* ARG (PDB ID: 4ITY) as a template in the SWISS-MODEL online server ^40^ and afterwards we determined the protonation/deprotonation states at pH 2.0 and pH 7.0 in the PDB2PQR. ^45^ Since ARG is a trimeric metalloprotein with three active sites binding to two manganese atoms (Mn ^+2^), we fixed the Mn ^+2^ coordination with active site residues and a hydroxyl molecule (OH ^−1^), considering the following coordination: first Mn ^+2^ with His114 (ND1), ASP137 (OD2), ASP141 (OD2), ASP243 (OD2) and the second Mn ^+2^ with ASP137 (OD1), HIS139 (ND1), ASP243 (OD1) and ASP245 (OD2). The MD simulation was reproduced in GROMACS v. 2020 ^46^ (RRID:SCR_014565), considering the AMBER99 ^47^ force field. The systems were solvated with the TIP3P water model, and Na ^+1^ or Cl ^−1^ ions were added for neutralization. The box size was 12×12×12 nm. Thus, the energy minimization was performed with the steep-descent algorithm with 20000 steps of calculation. The MD simulation was done in two steps; the first step was in the canonical ensemble (NVT) considering distance restraint of Mn ^+2^ to the active site by 5 ns. The second step was the MD production in the isothermal-isobaric ensemble (NPT) with a time of 100 ns. The V-rescale ^48^ thermostat was used to regulate the temperature at 309.65 K and the Parrinello-Rahman barostat at a reference pressure of 1 bar. Molecular docking was done with the DockThor online server ^49^; in the last frame, the molecular docking at two pH conditions was used as a receptor. A grid was considered in the active site of ARG (ChainA). The complex models with the best scores were chosen, and these were subsequently simulated in the isothermal-isobaric ensemble NPT for 100 ns. Gibbs free energy was calculated by the molecular mechanics-generalized Born surface area (MM-GBSA) ^50^ method in gmx_MMPBSA tool based on AMBER’s MMPBSA.py, and AmberTools20 ^51^ (RRID:SCR_014565) package was used. Additionally, to compare the binding free energy studies, we include the free energy perturbation (FEP) analysis where the Bennett acceptance ratio (BAR) calculates the free energy differences. ^52^ This analysis is achieved with the free energy implementation by the GROMACS tool.

### Statistical analysis

Results were entered into Microsoft Excel (version 10.0, Microsoft Corporation, Redmond, WA, USA) spreadsheets and analyzed by GraphPad Prism version 9.4.0 for Windows, GraphPad Software, San Diego, California USA, ( http://www.graphpad.com) (RRID:SCR_002798). To evaluate the correlation between the binding affinities of the compounds against the protein targets, they were placed in a linear regression plot and analyzed by Pearson’s correlation coefficient; differences were considered significant when p<0.05. Further, the selectivity score of binding affinities was calculated as described ^53^; where a selectivity value >1 indicates a priority of the compounds to bind to the parasite ARG over the human target. Heatmaps were constructed in the R programming environment (version 4.0.3) using the “heatmap 2” function in the package “gplots”. ^54^

## Results

### Data collection and virtual screening

In this work, a search was performed in the NuBBEDB for NPs that had been described with anti-leishmanial and anti-ARG activities. The search in the database resulted in 33 NPs described with anti-leishmanial activity, whereas six of them had also been described as inhibitors of ARG activity. Startlingly, all the NPs selected were described in the same article, in which the compounds were isolated from *Byrsonima coccolobifolia* species and tested for *in vitro* anti-ARG activity. ^55^ Since no anti-leishmanial activity was reported in the article, a cross-reference search for each compound was performed in the PubMed database to validate the properties. Thereafter, the SMILES from quercetin (NuBBE_122), isoquercetin (NuBBE_123), quercitrin (NuBBE_161), (+)-syringaresinol (NuBBE_214), catechin (NuBBE_287) and (-)-epicatechin (NuBBE_866) were obtained from PubChem and submitted to physicochemical properties analysis related to an absorption, distribution, metabolism, and excretion (ADME) profile; Lipinski’s rule of five (MW, iLOGP, HBAs and HBDs), ^56^ the quantitative estimate of drug-likeness (TPSA, RB, NHA and the number of alerts for undesirable substructures) ^57^ and the synthetic accessibility, ^58^ of the NPs are shown in Table 1.

**Table 1.** Natural compounds description selected in the NuBBE database.

NuBBE ID	PubChemID	Name	MW	iLOGP	TPSA	HBA	PAINS	Brenk	SynAcce
NuBBE_122	5280343	Quercetin	302.240	1.630	131.36	7	1	1	3.230
NuBBE_123	5378597	Isoquercetin	464.380	2.110	210.51	12	1	1	5.320
NuBBE_161	5353915	Quercitrin	448.380	1.270	190.28	11	1	1	5.280
NuBBE_214	100067	(+)-Syringaresinol	418.440	3.520	95.84	8	0	0	4.360
NuBBE_287	1203	Catechin	290.270	1.470	110.38	6	1	1	3.500
NuBBE_866	72276	(-)-epicatechin	290.270	1.470	110.38	6	1	1	3.500

MW: Molecular weight; iLOGP: octanol/water partition coefficient; TPSA: topological polar surface area; HBA: number of H-bond acceptors; HBD: Number of H-bond Donors; RB: rotatable bonds; NHA: number of heavy atoms; SynAcce: synthetic accessibility.

**Figure 1.**
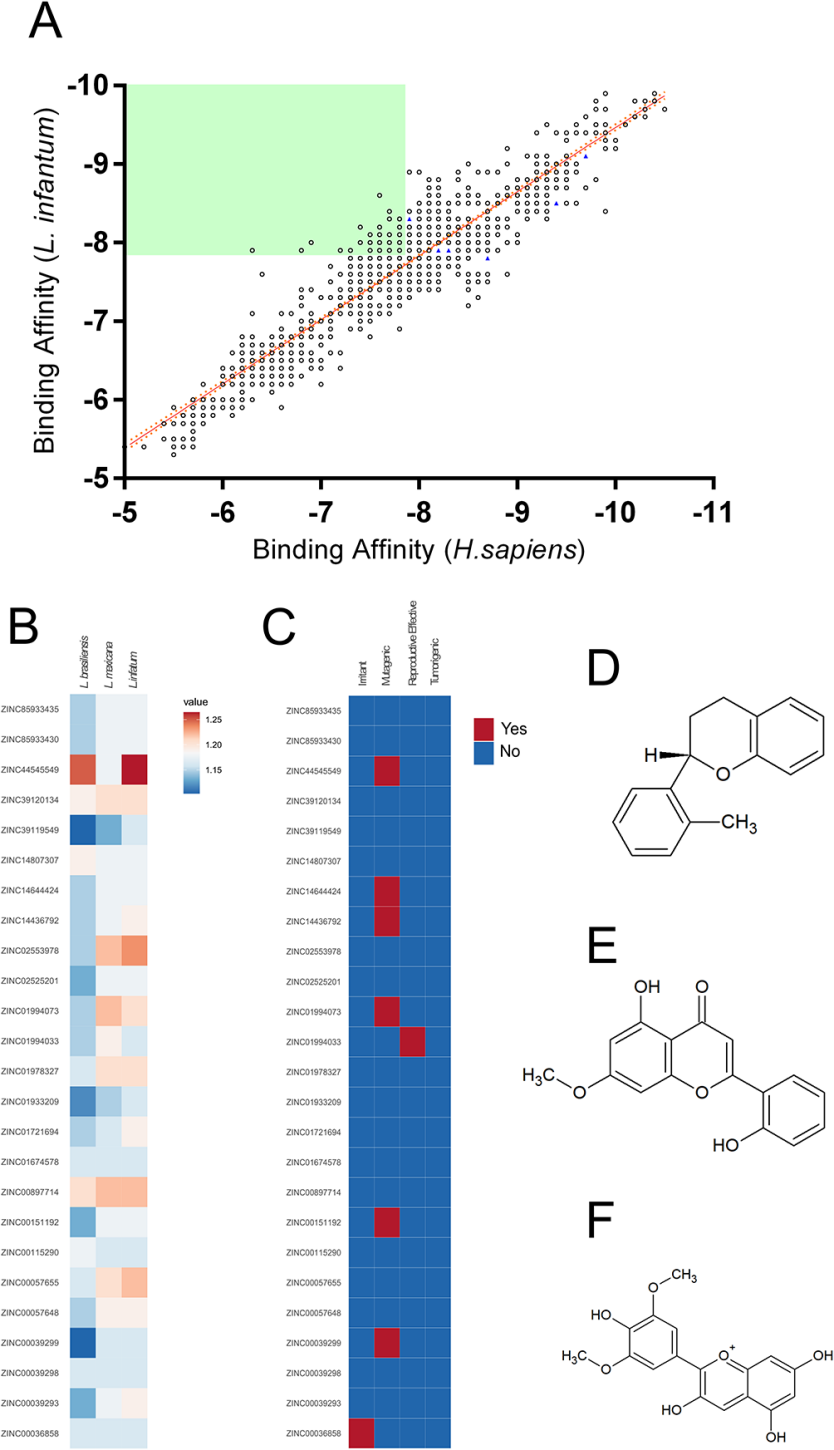
Virtual screening of the compounds selected from the NuBBE database. Binding affinities toward *L. infatum* and *H. sapiens* ARG targets were analyzed by linear regression and Pearson’s correlation coefficient. Solid orange line: linear regression; dotted orange lines: 95% confidence intervals. The solid green square was calculated using the maximum binding affinities of the 6 NPs (A). Normalized binding affinities heatmap of 25 selected compounds on *L. infantum*, *L. mexicana*, and *L. braziliensis* against their human homolog (B). Binary heatmap showing positive (red) or negative (blue) predicted toxicities (C). Chemical structure of ZINC39120134 (D), ZINC14807307 (E), and ZINC897714 (F).

To find structural analogs to the six NPs selected, a search of the SwissSimilarity server employing the commercial zinc-drug like compound library was performed, resulting in 400 analogs for each NP; however, the search comprised a high degree of redundancy between the analogs and a step in which duplicated compounds were excluded was executed, resulting in a total of 1499 unique compounds selected for virtual screening ( Figure 1). The virtual screening results against *Leishmania infantum* and human ARG, which shown a 44% of sequence identity, are plotted in Figure 1A, where a positive linear relationship between the binding affinities of the compounds toward both targets is shown [Pearson r:0.931; r2:0.868]. Later, aiming to select compounds that showed higher affinity toward *L. infantum* ARG, the selectivity was calculated, and compounds with scores >1 were screened, resulting in 25 compounds selected ( Figure 1A). Since *in vitro* evidence of inter-species differences in the susceptibility of parasites to anti-leishmanial drugs has been reported, ^59^ putative drug candidates must be active against several species of the parasite ^60^; in this way, the selectivity of the compounds against *L. mexicana* and *L. braziliensis* ARG were also calculated and plotted in a heatmap; each compound’s results showed differences in their affinities profile ( Figure 1B). Also, to select potential nontoxic candidates, the tumorigenic, mutagenic and reproductive effects, as well as irritant action were assessed for the 25 compounds ( Figure 1C). Thus, the compounds 2H-1-benzopyran, 3,4-dihydro-2-(2-methylphenyl)- (9CI) (ZINC39120134) ( Figure 1D), echioidinin (ZINC14807307) ( Figure 1E), and malvidin (ZINC897714) ( Figure 1F) were selected for further analysis, since they showed favorable binding affinities against the three parasite species targets and negative results for potential toxicities.

### Molecular dynamics simulations (MDS)

**Figure 2.**
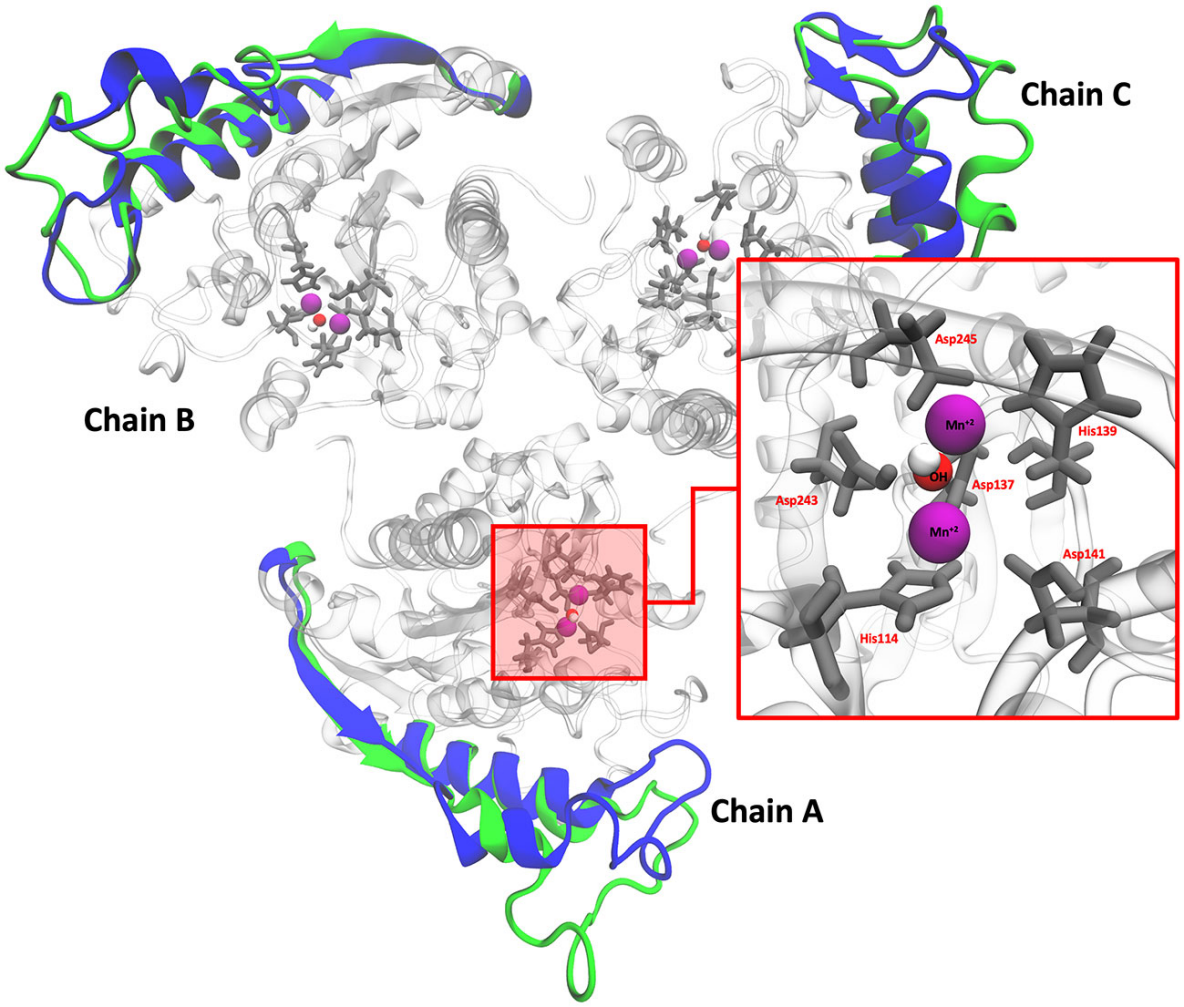
Structural conformation of ARG with its active site. Colors blue and green represent the cartoon representation of pH 2.0 and pH 7.0. The red box shows the active site of ARG.

**Figure 3.**
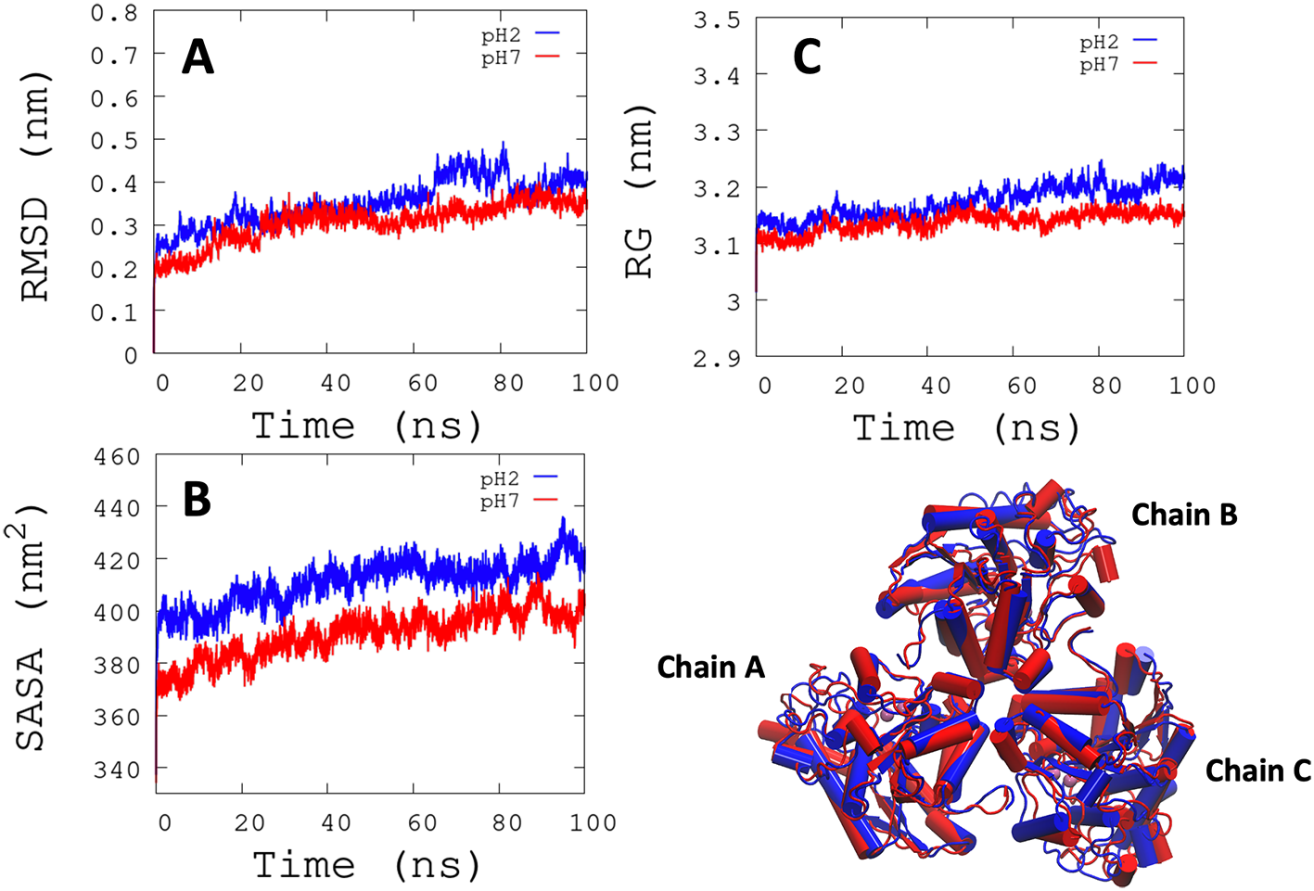
RMSD, SASA, and RG analysis. (A) RMSD is shown the conformational changes reported at pH 2.0. (B) SASA shows a greater solvent access surface area to ARG at pH 2.0 than at pH 7.0. (C) RG shows the same behavior as RMSD.

**Figure 4.**
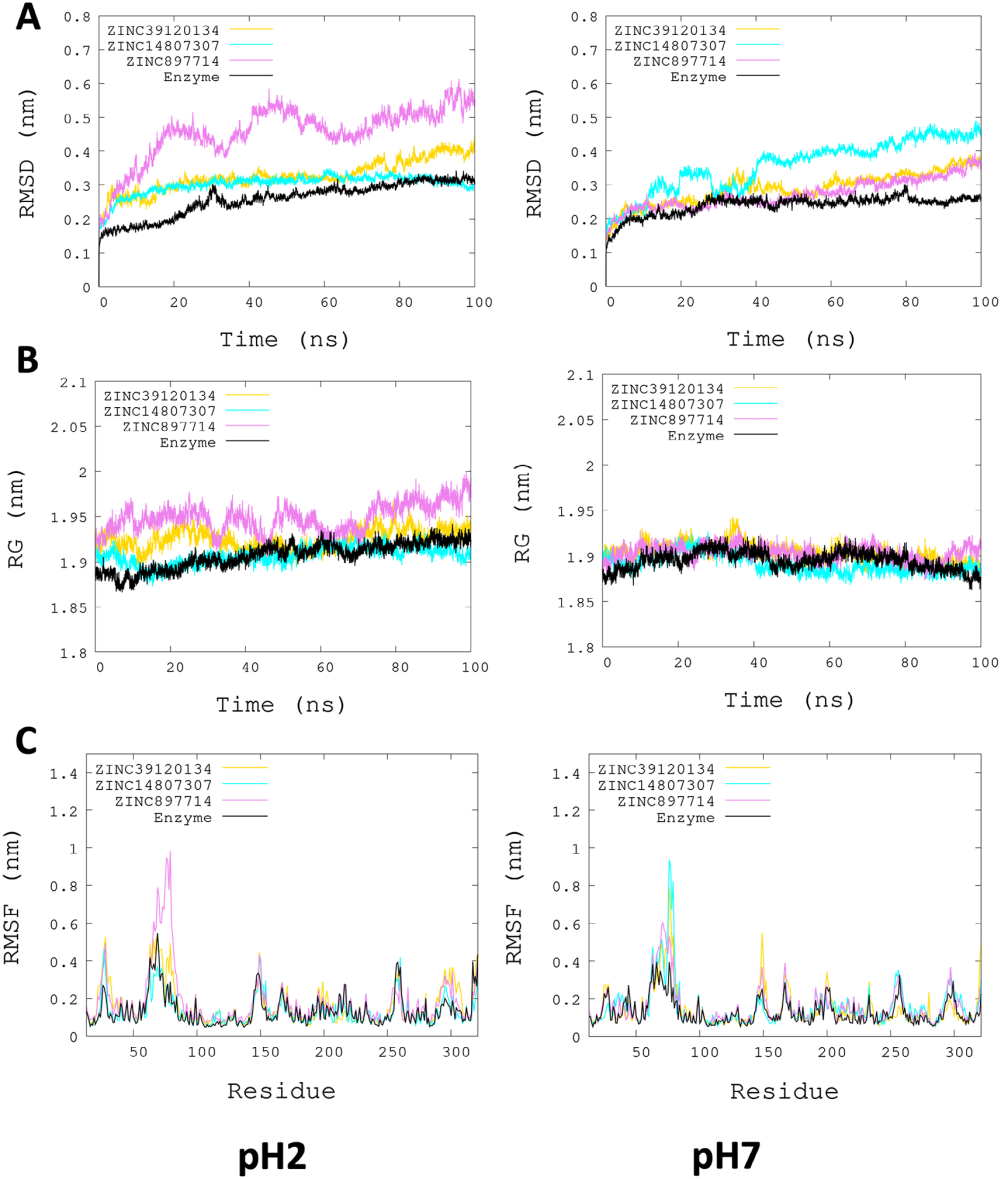
Plots of MD simulation of each complex. More significant conformational changes of ARG enzyme are shown at pH 2.0. (A) RMSD plot of ChainA concerning the whole protein. (B) RG analysis. (C) RMSF *per residue* of backbone.

**Figure 5.**
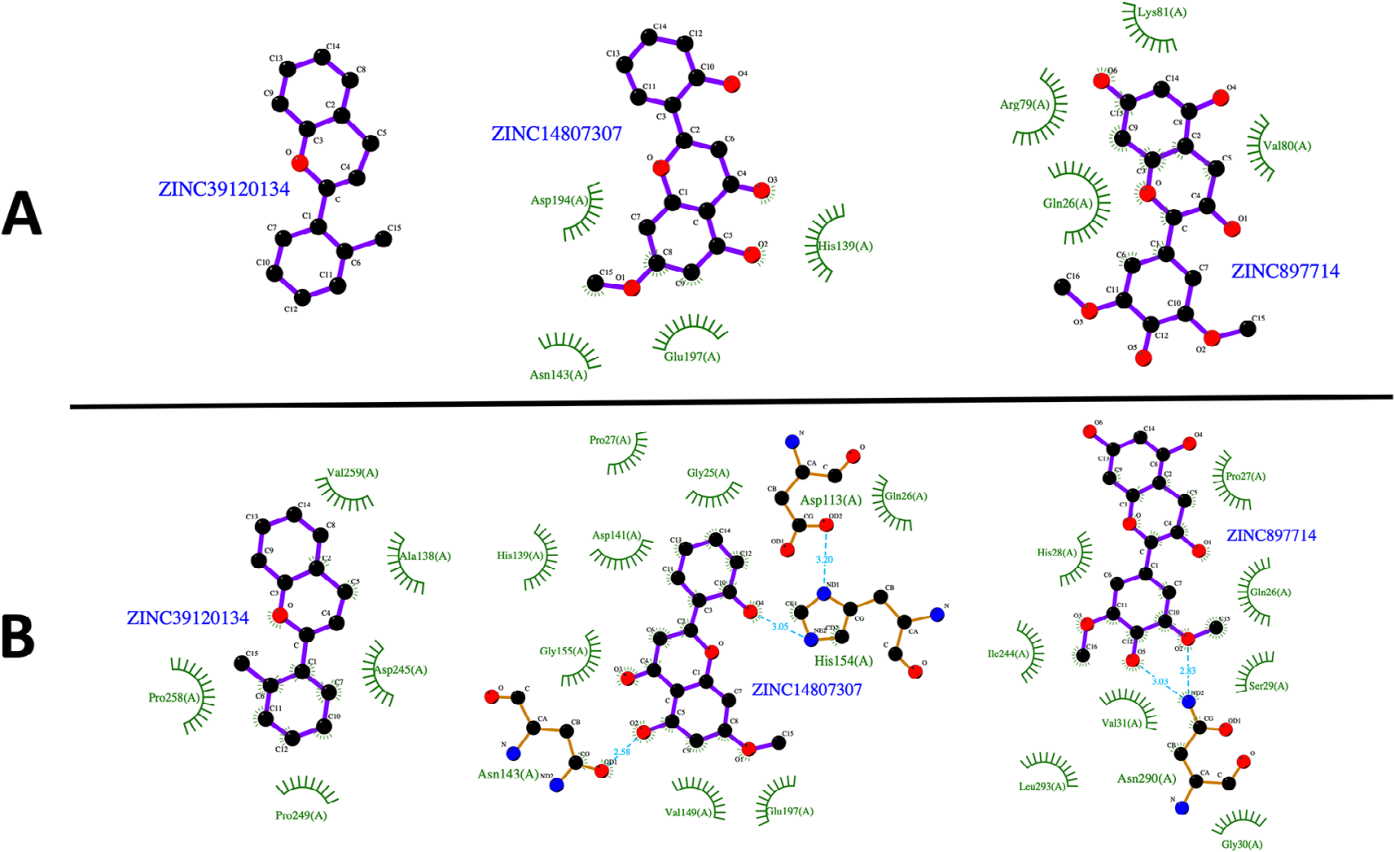
2D representation of the last frame of each complex. (A) Last frame at pH 2.0. and (B) Last frame at pH 7.0. Green represents the hydrophobic interaction between enzyme-ligand, color sky blue represents the hydrogen bond interaction.

*L. infantum* ARG is an enzyme with trimeric conformation (ChainA, ChainB, and ChainC) and its structure showed stable behavior during a 100 ns of MDS performed at pH 2.0 and pH 7.0 ( Figure 2). Here we included the metal ions (Mn ^+2^) and one hydroxyl molecule (OH ^−1^) for each active site, and it was observed that some regions lose their structural conformation at pH 2.0 conditions (green color). In addition, compared to ARG at pH 7.0, ARG at pH 2.0 exhibits large structural alterations and high variations per residue (see Figure 3A and 3B). In Figure 3C, the radius of gyration shows lower compaction of whole protein during the MDS at pH 7.0 than at pH 2.0. The report of the trajectory of each complex system (enzyme-ligand) and the protein without ligand is shown in Figure 4. Since the root-mean-squared deviation (RMSD) is a noteworthy analysis to verify the similarity between a protein-bound and not bound ligand. ^61^ The RMSD values in nm are presented that were taken from the ChainA of each protein in different pH conditions, whereas the enzyme-ligand systems presented greater conformational changes in the substrate-binding site ( Figure 4A). Likewise, radius of gyration (RG) analysis verifies the compactness of protein structures, where the lowest RG demonstrates the tightest packing and high conformational stability. ^62^ The results showed that, at pH 2.0, low compactness and a large broadening of the macromolecules are reported ( Figure 4B). Figure 4C shows the root-mean-squared fluctuation (RMSF) *per residue* of the backbone, where high fluctuations were shown from residue 50 to 100 in both systems. From the enzyme-ligand simulation results, we take each simulation’s last frames ( Figure 5). The compounds ZINC14807307 and ZINC897714 generate exciting interactions in the active center at the pH conditions evaluated and, at pH 7.0, hydrogen bonds are observed, which benefits enzyme-ligand coupling.

### Binding free energy estimation

The binding free energy analysis of pH 2.0 and pH 7.0 from the frames of each simulation is shown in Table 2. The propitious energetic contribution with a binding free energy of -28.59 kcal/mol (ZINC897714/pH2) maximum and -14.07 kcal/mol (ZINC14807307/pH2) minimum were obtained. The estimated phase-gas binding free energy (ΔGgas) provided the highest energy contributions for ZINC897714 in both pHs. Contrary, the van der Waals energies (ΔEvdW) provided the highest energy contributions at pH 2.0 in ZINC897714.

**Table 2.** MM-GBSA binding free energy estimation average values.

Energy component	ZINC39120134		ZINC14807307		ZINC897714	
	pH2	pH7	pH2	pH7	pH2	pH7
ΔEvdW	-20.22	-23.65	-20.41	-23.62	-33.46	-32.06
ΔEele	-2.08	-3.93	-4.41	-5.24	-24.76	-18.38
ΔEgb	10.37	12.63	13.26	14.98	33.90	26.00
ΔEsurf	-2.62	-3.02	-2.51	-3.15	-4.26	-3.83
ΔGgas	-22.30	-27.58	-24.83	-28.86	-58.22	-50.44
ΔGsolv	7.75	9.62	10.75	11.02	29.64	22.18
ΔGTotal	-14.55	-17.96	-14.07	-17.04	-28.59	-28.26

ΔEvdW = Van Der Waals energy; ΔEele = electrostatic energy; ΔEgb = electrostatic contributionfree energy calculated by generalized Born; ΔGgas = estimated phase-gas binding free energy; ΔGsolv = estimates binding free energy solvent; ΔG TOTAL = estimated binding free energy. Values of energy in kcal/mol.

It is well understood that hydrophobic interactions favorably contribute to binding. The electrostatic energies (ΔEele) contributed positively to the binding enzyme-ligand, which the best energy was -24.76 kcal/mol (ZINC897714/pH2). Despite this, the solvation energies (ΔGsolv) offset the negative electrostatic interactions, thus unfavorably contributing to the binding of ZINC897714 to ARG in both pHs (ZINC897714/pH2 = 29.64 kcal/mol and ZINC897714/pH7 = 22.18 kcal/mol). These results show that the protonation states at a given pH can positively or negatively favor the enzyme-ligand binding, where it is expected that at a pH above 7.0 the enzyme-ligand binding can be increased.

In an attempt to improve the enzyme-ligand binding energy analysis, the FEP approach was used, which estimates the difference in free energy between two states ( *A* state and *B* state) by slowly change from one state to another. *A* state corresponds to the initial state of free energy and *B* state corresponds to the final state. This study sampled 20 microstates with a time of 20 ns for each microstate; the results are presented in Table 3. Herein, it is observed that, at both pHs, the best compounds occurred in the following order: ZINC14807307 > ZINC897714 > ZINC39120134. On the other hand, the compounds ZINC14807307 and ZINC897714 are shown to be stable at pH 2.0 conditions.

**Table 3.** FEP and MM-GBSA average values of ΔG TOTAL in kcal/mol.

Compound	FEP _***pH*2**_	FEP _***pH*7**_	MM-GBSA _***pH*2**_	MM-GBSA _***pH*7**_
ZINC39120134	3.38	4.77	-14.55	-17.96
ZINC14807307	-6.27	-4.08	-14.07	-17.04
ZINC897714	-2.80	-0.29	--28.59	-28.26

## Discussion

The World Health Organization (WHO) considers leishmaniasis to be one of the major neglected global diseases and responsible for millions of disability-adjusted life years (DALYs), representing one of the top burdens among the neglected tropical diseases. ^63^ Worldwide, 13 countries have a high burden of VL (Bangladesh, China, Ethiopia, Georgia, India, Kenya, Nepal, Paraguay, Somalia, South Sudan, Spain, Sudan, and Uganda), and 11 have a high burden of TL (Afghanistan, Algeria, Colombia, Iran, Morocco, Pakistan, Peru, Saudi Arabia, Syrian Arab Republic, Tunisia, and Turkey), while Brazil has a high burden of both clinical forms. ^64^ Thus, TL treatment choice is based on the clinical presentation and infecting species, while any person with VL signs and symptoms and a verified diagnosis warrants chemotherapy. ^65^ The range of currently available drugs for treating leishmaniasis is relatively small and it includes repurposed molecules, such as amphotericin B, miltefosine, and paromomycin; while few new drug candidates reached clinical trials in the last decades. ^66^^,^^67^ For these reasons, the investigation of new therapies has been very active recently, and a wide range of compounds have been identified as potential hits and leads. ^68^ The unique and vast chemical diversity of NPs places them as a major component of the biologically relevant chemical space, ^69^ while NP classes like alkaloids, coumarins, flavonoids, lignans, neolignans, quinones, and terpenoids have demonstrated anti-leishmanial activity. ^70^ Several of these that target *Leishmania* ARG have been investigated for their potential as new drug candidates, although quercetin, ^71^^–^^73^ catechin, (-)-epicatechin, (+)-syringaresinol, isoquercetin, quercitrin, resveratrol, and cinnamic acid derivatives had shown *in vitro* efficacy. ^31^^,^^33^^,^^74^ Additionally, certain NPs had demonstrated favorable *in vivo* effectivity, including epigallocatechin gallate, ^75^ gallic acid, ^76^ rosmarinic acid, ^77^ and quercetin. ^78^^,^^79^ The equilibrium between biological activity and pharmacological qualities is one of several aspects, nevertheless, that restricts the translation of NPs into commercial drugs. ^80^^,^^81^ *In silico* based drug repositioning potential for discovering new applications for existing drugs and for developing new drugs in pharmaceutical research and the industry has gained importance ^82^^,^^83^; whereas, in the chemical structure and molecule information approach, the structural similarity is incorporated with molecular activity and other biological information to identify new associations. ^84^

The present work aimed to apply CADD approaches to select analogs to NPs with known anti-leishmanial and anti-ARG activities; although results of the quercetin analogs, the anthocyanin malvidin (ZINC897714; PubChem CID: 159287), and the flavone echioidinin (ZINC14807307; PubChem CID: 15559079) showed favorable binding affinity to *L. infantum*, *L. mexicana*, and *L. braziliensis* ARG and no predicted toxicity. Besides that, in the ARG super-family, the active site is conserved in all organisms, which includes the coordination of divalent metal Mn ^2+^, ^85^ and differences between the parasite and its human homolog have been described, ^86^^,^^87^ highlighting the possibility to target selectively the parasite enzyme. However, recently, cinnamides ^88^ and 1-phenyl-1H-pyrazolo[3,4-d] pyrimidine synthetic derivatives ^89^ have been described as potential selective inhibitors of parasite ARG and have shown *in vitro* anti-leishmanial activity. A major bottleneck of drug discovery for leishmaniasis was aimed at the *in silico* workflow proposed, which is that compounds must show activity in the acidic environment of the phagolysosome ^90^; thus, the analyzed compounds in this work showed stable enzyme-ligand interaction and favorable binding free energy at pH 2.0 in MDS analysis. However, when taking into consideration the target product profile (TPP), proposed by the Drugs for Neglected Diseases initiative (DNDi), which includes regard for the oral route of administration for new candidates, ^91^ both ADME profiles showed the potential for oral route administration and high bioavailability, but only malvidin results have been ratified by experimental studies published elsewhere. ^92^^–^^94^ Furthermore, malvidin has shown the potential to be an antioxidant, anti-hypertensive, anti-inflammatory, anti-obesity, anti-osteoarthritis, anti-proliferative, and anticancer drug candidate, ^95^^–^^99^ whereas to the best of our knowledge no research has been published studying the potential pharmacological activity of echioidinin. Anthocyanins are commonly found in many plants, while the most common types are cyanidin, delphinidin, pelargonidin, peonidin, petunidin, and malvidin, which are distributed in fruits and vegetables in 50%, 12%, 12%, 12%, 7%, and 7% proportions, respectively. ^100^ These molecules are more stable at a lower pH solution, and in such conditions the flavylium cation formed enables the anthocyanin to be highly soluble in water. ^101^ The physicochemical properties offered by anthocyanins should be considered of interest for anti-leishmanial drug discovery since the parasite is adapted to live in parasitophorous vacuoles of infected macrophages in mammalian hosts, where it survives, proliferates, and is responsible for the development of the active disease. ^102^ Recently, the anthocyanidin profile of *Arrabidaea chica* has been examined and its anti-leishmanial activity analyzed, ^103^ and carajurin (PubChem CID: 44257040) showed the highest activity against the intracellular parasites, altering all parameters of *in vitro* infection. ^104^ Additionally, it has been shown that carajurin leads to a decrease in the mitochondrial membrane potential, an increase in ROS production, and cell death by late apoptosis in *L. amazonensis.* ^105^ Furthermore, flavones showing anti-leishmanial potential have been described in the literature, ^106^ whereas apigenin (PubChem CID: 5280443) and luteolin (PubChem CID: 5280445) have shown the potential of inhibiting the growth of *L. amazonensis.* ^107^

Limitations of the present study should be also mentioned, such as the protein dynamics and complex stabilities with MDS lasting within nanoseconds scales (0-100 ns), while most structural dynamics and biological activities of proteins occur within timescales of microseconds and milliseconds. ^108^ Even so, complex dynamics and interactions between enzymes and ligands have been reported using nanosecond timescales. ^109^^,^^110^ Additionally, the work did not include *in vitro* or *in vivo* validation. It is important to note that anti-leishmanial *in vitro* assays have drawbacks, including metabolic differences between the amastigote and promastigote stages, ^111^ variations in drug effectiveness and susceptibility among parasites isolated from patients, ^112^ and a variety of biochemical pathways linked to drug-resistant phenotypes in the parasite, ^113^^,^^114^ which can lead to false positive results. Additionally, numerous animal models are used in the validation tests for VL and TL drug candidates; however, due to insufficient translation to human disease, their predictive value is frequently low. Furthermore, reliable main models for VL are frequently employed, including Syrian golden hamsters and BALB/c mice, ^115^^,^^116^ while there are no validated animal models for TL since different species experience varied clinical symptoms, and current models lack human characteristics such as pathophysiology, symptomatology, and treatment response. ^117^

## Conclusion

In the first screening, this work identified three substances with natural products structural analogs with potential effects against *Leishmania* ARG using *in silico* analysis from the available data and research of natural products found in databases. The substances were: ZINC39120134 (3,4-dihydro-2-(2-methylphenyl)-(9CI)), ZINC14807307 (echioidinin) and ZINC897714 (malvidin), where the most suitable compounds were ZINC14807307 and ZINC897714, showing favorable binding affinity to *L. infantum*, *L. mexicana*, and *L. braziliensis* ARG, no potential toxicity and stability at pH 2.0; important factors due to the acidic environment of the phagolysosomes of mammalian hosts. Taking into consideration that the oral bioavailability of malvidin has experimental data published and that its pharmacological potential has been widely studied, the results presented in this work warrant further *in vitro* and *in vivo *studies using malvidin to confirm its potential as a drug candidate against leishmaniasis.

## Data Availability

Figshare. Supplementary material. https://figshare.com/articles/dataset/Supplementary\_material\_xlsx/21867822. ^118^ This project contains the following underlying data:
•
Table S1. (Compounds obtained by chemical similarity against the natural products analyzed) •
Table S2. (Virtual screening results of the compounds selected against *L. infatum* and *H. sapiens*arginase enzymes) •
Table S3. (Virtual screening results of the compounds selected against *L. braziliensis* and *L. mexicana* arginase enzymes) •
Table S4. (Toxicity prediction of the selected compounds) Data are available under the terms of the Creative Commons Zero “No rights reserved” data waiver (CC0 1.0 Public domain dedication).
