# Supplementary material for: Computer-aided drug design approaches applied to screen natural product’s structural analogs targeting arginase in Leishmania spp
Source: F1000Res. 2023 Jul 13;12:93. Originally published 2023 Jan 24. [Version 3] doi: 10.12688/f1000research.129943.3 (PMC10323282; doi:10.12688/f1000research.129943.3)
Supplement: Peer review history

# Peer review: Reviewer response for version 3

**DOI:** 10.5256/f1000research.152921.r187098

**Published:** 2023-07-31

**Authors:** Anupam Nath Jha

**Affiliations:** 1 Tezpur Central University, Tezpur, India

Authors are modelling the structure of Homo sapiens ARG's whereas the experimental structures are available for the same. Any reason?

Is the work clearly and accurately presented and does it cite the current literature?

Partly

If applicable, is the statistical analysis and its interpretation appropriate?

Not applicable

Are all the source data underlying the results available to ensure full reproducibility?

Partly

Is the study design appropriate and is the work technically sound?

Partly

Are the conclusions drawn adequately supported by the results?

Partly

Are sufficient details of methods and analysis provided to allow replication by others?

No

Reviewer Expertise:

NA

I confirm that I have read this submission and believe that I have an appropriate level of expertise to confirm that it is of an acceptable scientific standard.

# Peer review: Reviewer response for version 3

**DOI:** 10.5256/f1000research.152921.r187097

**Published:** 2023-07-21

**Authors:** Rubens L. Monte-Neto, Alessandra Sousa

**Affiliations:** 1 Biotechnology Applied to Pathogens (BAP), Instituto René Rachou, Fundação Oswaldo Cruz (FIOCRUZ), Belo Horizonte, Brazil; 2 Biotechnology Applied to Pathogens (BAP), Instituto René Rachou, Faculdades Oswaldo Cruz, São Paulo, State of São Paulo, Brazil

Although authors provided the 3rd version of the manuscript following reviewer #2 suggestions, there is no rebuttal letter on the queries stated by the 3rd reviewer, which I am including again here

 Authors used computer-aided tools to better understand anti-leishmanial activity of natural products-based analogs toward arginase binding. Based on 33 anti-leishmanial NPs, authors selected 6 compounds able to inhibit ARG; selected 1499 analogs and filtered 25 compounds by selectivity and bonding affinity towards ARG from Leishmania. Three out of 25 analogs were further investigated and authors affirm they are good hits to be validated. The rational is quite clear and the manuscript well organized. However, several points must be addressed in order to improve impact and paper quality

 Comments

-  Authors initially selected 33 anti-leishmanial natural products on NuBBE database. More than one year after their access I’ve got the same 33 selected NPs with anti-leishmanial properties, which I think is very limited. Some of anti-leishmanial NPs I know were not listed there. This must be highlighted as one of the study limtations. Several other molecules are missing which would improve manuscript impact.
- Page 4 (1st paragraph): I suggest the authors to rerun ADME analysis on pkCSM tool, considering ADMET https://biosig.lab.uq.edu.au/pkcsm/
- Page 4 (2nd paragraph): I understand that SWISS-MODEL can be a very useful tool. However ,some limitations could impact on assertiveness. Considering our recent advances on modelling, I encourage authors to consider AlphaFold and after comparing with Swiss-model results highlight any study limitations or confirm that swiss-model was enough to reach the main goal. https://www.nature.com/articles/s41586-021-03819-2
- Page 4 (3rd paragraph): "The Osiris Data Warrior software... and reproductive effects" In order to improve toxicity prediction, not only based in a fragment library, I strongly encourage authors to perform analysis using graph-based tools. Please find below a link were several of these tools could be used. For example cardioToxCSM, embryoToxCSM, pdCSM-PPI, toxCSM, mCSM-lig… https://biosig.lab.uq.edu.au/tools
- Page 4 (4th paragraph): Why authors considered pH 2.0 (acidic), once the parasitophorous vacuole is around pH 4.7-5.2? I suggest that authors consider more realistic pH inspired by real measures of Leishmania PV https://pubmed.ncbi.nlm.nih.gov/1689700/#:~:text=We%20found%20statistically%20different%20mean,to%205.26%20for%20parasitophorous%20vacuoles.; https://link.springer.com/article/10.1007/s40495-020-00209-6
- Page 5 (3rd paragraph): "...all compounds with scores... in 25 compounds selected" Please provide a table showing all selectivities (>1) to the selected compounds. Fig 1B is showing binding affinities but we need to see the relationship with selectivity
- Page 5 (3rd paragraph): "Thus the compounds.. potential toxicities" This is partially true. When carefully analyzing Fig 1B, I agree with the authors that compounds ZINC897714 and ZINC39120134 showed acceptable binding affinities among the three testes Leishmania species. However, compound ZINC14807307 presented divergent binding affinities when comparing the three tested species. Additionally, compound ZINC44545549 presented the bests binding affinities - with comparable BA considering ZINC..7307 for L. mexicana - although it was not included for further analysis. Based on that, I suggest to authors to consider ZINC44545549 for the pipeline.
- Page 5 (4th paragraph): "... it was observed..(green color)" What about pH 4-5, which match with Leishmania PV
- Fig 1: Instead of predicting only 4 toxicity parameters, authors are encouraged to run pkCSM (graph-based ADMET prediction experimentally curated) and predict:
  ABSORPTION: water solubility, Caco2 permeability, Instestinal abssorption, skin permeability, P-glycoprotein substrate, P-glycoprotein I inhibition, P-glycoprotein II inhibition
  DISTRIBUTION: VDss (human), Fraction unbound (human), BBB permeability, CNS, permeability
  METABOLISM:, CYP2D6 substrate, CYP3A4 substrate, CYP1A2 inhibitor, CYP2C19 inhibitor, CYP2C9 inhibitor, CYP2D6 inhibitor, CYP3A4 inhibitor
  EXCRETION: Total clearance, Renal OCT2 substrate
  TOXICITY: AMES toxicity, Max tolerated dose (human), hERG I inhibitor, hERG II inhibitor, Oral Rat acute toxicity (LD50), Oral rat chronic toxicity (LOAEL), Hepatotoxicity, Skin sensitization, T. pyriformis toxicity, Minnow toxicity
  All parameters can be show as binary heatmap (as in Fig 1B) including hierarchical clustering to better visualization
- Page 10 (1st paragraph): "ZINC14807307...pH 2.0 conditions" Please consider ZINC44545549 for this conclusion!
- Page 10 (3rd paragraph): "...showed favorable.. no predicted toxicity" Toxicity prediction in the work is very poor and must be reinvestigated. Selected compounds (25) showed the highest binding affinity around 1.25 (Fig. 2B). Authors must provide what it represents in terms of selectivity. Reference anti-leishmanial candidates such as miltefosine and amphotericin B, show SI of hundreds. This is a study limitation and additional experimental validation are required to make predictions trustable

Minor

- Page 3 (2nd paragraph): "...been described with leishmanicidal..." Please confirm that in references 18-21 we found indeed leishmanicidal agents, otherwise, replace the term leishmanicidal by anti-leishmanial 
- Page 3 (2nd paragraph): "..apoptosis." Please replace by programmed cell death.. https://pubmed.ncbi.nlm.nih.gov/23202528/
- Fig 1: Font size within Fig 1 should be reduced. They are too big compared to the other fonts
- First paragraph of discussion is introduction and should be removed, since the main theoretical basis was already provided in introduction section

Is the work clearly and accurately presented and does it cite the current literature?

Partly

If applicable, is the statistical analysis and its interpretation appropriate?

Partly

Are all the source data underlying the results available to ensure full reproducibility?

No

Is the study design appropriate and is the work technically sound?

Yes

Are the conclusions drawn adequately supported by the results?

No

Are sufficient details of methods and analysis provided to allow replication by others?

Yes

Reviewer Expertise:

Molecular parasitology, anti-leishmanial compounds, pharmacology

We confirm that we have read this submission and believe that we have an appropriate level of expertise to confirm that it is of an acceptable scientific standard, however we have significant reservations, as outlined above.

# Peer review: Reviewer response for version 2

**DOI:** 10.5256/f1000research.148092.r178761

**Published:** 2023-07-05

**Authors:** Rubens L. Monte-Neto, Alessandra Sousa

**Affiliations:** 1 Biotechnology Applied to Pathogens (BAP), Instituto René Rachou, Fundação Oswaldo Cruz (FIOCRUZ), Belo Horizonte, Brazil; 2 Biotechnology Applied to Pathogens (BAP), Instituto René Rachou, Faculdades Oswaldo Cruz, São Paulo, State of São Paulo, Brazil

Authors used computer-aided tools to better understand anti-leishmanial activity of natural products-based analogs toward arginase binding. Based on 33 ant-leishmanial NPs, authors selected 6 compounds able to inhibit ARG; selected 1499 analogs and filtered 25 compounds by selectivity and bonding affinity towards ARG from Leishmania. Three out of 25 analogs were further investigated and authors affirm they are good hit to be validated. The rational is quite clear and the manuscript well organized. However, several points must be addressed in order to improve impact and paper quality

 Comments

-  Authors initially selected 33 anti-leishmanial natural products on NuBBE database. More than one year after their access I’ve got 33 selected NPs with anti-leishmanial properties, which I think is very limited. Some of anti-leishmanial NPs I know, were not listed there. This must be highlighted as one of the study limtations. Several other molecules are missing which would improve manuscript impact.
- Page 4 (1st paragraph): I suggest the authors to rerun ADME analysis on pkCSM tool, considering ADMET https://biosig.lab.uq.edu.au/pkcsm/
- Page 4 (2nd paragraph): I understand that SWISS-MODEL can be a very useful tool. However some limitations could impact on assertiveness. Considering our recent advances on modelling, I encourage authors to consider AlphaFold and after comparing with Swiss-model results highlight any study limitations or confirm that swiss-model was enough to reach the main goal. https://www.nature.com/articles/s41586-021-03819-2
- Page 4 (3rd paragraph): "The Osiris Data Warrior software... and reproductive effects" In order to improve toxicity prediction, not only based in a fragment library, I strongly encourage authors to perform analysis using graph-based tools. Please find below a link were several of these tools could be used. For example cardioToxCSM, embryoToxCSM, pdCSM-PPI, toxCSM, mCSM-lig… https://biosig.lab.uq.edu.au/tools
- Page 4 (4th paragraph): Why authors considered pH 2.0 (acidic), once the parasitophorous vacuole is around pH 4.7-5.2? I suggest that authors consider more realistic pH inspired by real measures of Leishmania PV https://pubmed.ncbi.nlm.nih.gov/1689700/#:~:text=We%20found%20statistically%20different%20mean,to%205.26%20for%20parasitophorous%20vacuoles.; https://link.springer.com/article/10.1007/s40495-020-00209-6
- Page 5 (3rd paragraph): "...all compounds with scores... in 25 compounds selected" Please provide a table showing all selectivities (>1) to the selected compounds. Fig 1B is showing binding affinities but we need to see the relationship with selectivity
- Page 5 (3rd paragraph): "Thus the compounds.. potential toxicities" This is partially true. When carefully analyzing Fig 1B, I agree with the authors that compounds ZINC897714 and ZINC39120134 showed acceptable binding affinities among the three testes Lesihamnia species. However, compound ZINC14807307 presented divergent binding affinities when comparing the three tested species. Additionally, compound ZINC44545549 presented the bests binding affinities - with comparable BA considering ZINC..7307 for L. mexicana - although it was not included in further analysis. Based on that I suggest to authors to consider ZINC44545549 for the pipeline.
- Page 5 (4th paragraph): "... it was observed..(green color)" What about pH 4-5, which match with Leishmania PV
- Fig 1: Instead of predicting only 4 toxicity parameters, authors are encouraged to run pkCSM (graph-based ADMET prediction experimentally curated) and predict:
  ABSORPTION
  - water solubility
  - Caco2 permeability
  - Instestinal abssorption
  - skin permeability
  - P-glycoprotein substrate
  - P-glycoprotein I inhibition
  - P-glycoprotein II inhibition
  DISTRIBUTION 
  - VDss (human)
  - Fraction unbound (human)
  - BBB permeability
  - CNS permeability
  METABOLISM
  - CYP2D6 substrate
  - CYP3A4 substrate
  - CYP1A2 inhibitor
  - CYP2C19 inhibitor
  - CYP2C9 inhibitor
  - CYP2D6 inhibitor
  - CYP3A4 inhibitor
  EXCRETION
  - Total celarance
  - Renal OCT2 substrate
  TOXICITY
  - AMES toxicity
  - Max tolerated dose (human)
  - hERG I inhibitor
  - hERG II inhibitor
  - Oral Rat acute toxicity (LD50)
  - Oral rat chronic toxicity (LOAEL)
  - Hepatotoxicity
  - Skin sensitization
  - T. pyriformis toxicity
  - Minnow toxicity
  All parameters can be show as binary heatmap (as in Fig 1B) including hierarchical clustering to better visualization
- Page 10 (1st paragraph): "ZINC14807307...pH 2.0 conditions" Please consider ZINC44545549 for this conclusion!
- Page 10 (3rd paragraph): "...showed favorable.. no predicted toxicity" Toxicity prediction in the work is very poor and must be reinvestigated. Selected compounds (25) showed the highest binding affinity around 1.25 (Fig. 2B). Authors must provide what it represents in terms of selectivity. Reference antileishmanial candidates such as miltefosine and amphotericin B, show SI of hundreds. This is a study limitation and additional experimental validation are required to make predictions trustable

Minor

- Page 3 (2nd paragraph): "...been described with leishmanicidal..." Please confirm that in references 18-21 we found indeed leishmanicidal agents, otherwise, replace the term leishmanicidal by anti-leishmanial 
- Page 3 (2nd paragraph): "..apoptosis." Please replace by programmed cell death.. https://pubmed.ncbi.nlm.nih.gov/23202528/
- Fig 1: Font size within Fig 1 should be reduced. They are too big compared to the other fonts
- First paragraph of discussion is introduction and should be removed, since the main theoretical basis was already provided in introduction section

This review was initially made by one of our PhD candidates, in which I agree with the highlighted points and selected the major concerns for the final version of this review

Is the work clearly and accurately presented and does it cite the current literature?

Partly

If applicable, is the statistical analysis and its interpretation appropriate?

Partly

Are all the source data underlying the results available to ensure full reproducibility?

No

Is the study design appropriate and is the work technically sound?

Yes

Are the conclusions drawn adequately supported by the results?

No

Are sufficient details of methods and analysis provided to allow replication by others?

Yes

Reviewer Expertise:

Molecular parasitology, anti-leishmanial compounds, pharmacology

We confirm that we have read this submission and believe that we have an appropriate level of expertise to confirm that it is of an acceptable scientific standard, however we have significant reservations, as outlined above.

## References

[1] : pkCSM: Predicting Small-Molecule Pharmacokinetic and Toxicity Properties Using Graph-Based Signatures. *J Med Chem* .2015;58(9) : 10.1021/acs.jmedchem.5b00104 4066-72 10.1021/acs.jmedchem.5b00104 25860834PMC4434528

[2] : Highly accurate protein structure prediction with AlphaFold. *Nature* .2021;596(7873) : 10.1038/s41586-021-03819-2 583-589 10.1038/s41586-021-03819-2 34265844PMC8371605

[3] : Parasitophorous vacuoles of Leishmania amazonensis-infected macrophages maintain an acidic pH. *Infect Immun* .1990;58(3) : 10.1128/iai.58.3.779-787.1990 779-87 10.1128/iai.58.3.779-787.1990 1689700PMC258533

[4] : Survival Strategies of Leishmania Parasite: Too Many Questions and Few Answers. *Current Pharmacology Reports* .2020;6(2) : 10.1007/s40495-020-00209-6 25-27 10.1007/s40495-020-00209-6

[5] : Cell death in parasitic protozoa: regulated or incidental?. *Nat Rev Microbiol* .2013;11(1) : 10.1038/nrmicro2929 58-66 10.1038/nrmicro2929 23202528

# Peer review: Reviewer response for version 2

**DOI:** 10.5256/f1000research.148092.r178758

**Published:** 2023-07-04

**Authors:** Anupam Nath Jha

**Affiliations:** 1 Tezpur Central University, Tezpur, India

Authors have tried drug repurposing for Leishmaniasis and targeted arginase of *L. infantum*. There are some questions to be answered by authors:

1. Which pdb structure (pdb id) of human L-arginine was selected ( https://www.uniprot.org/uniprotkb/P05089/entry#structure) for docking?
2. What is the sequence & structure level similarity between human and *L. infantum* ARG proteins? Give the justification of selection for ARG protein as target for drug discovery based on that similarity.
3. Any reason for selection of two pH values (2 & 7) and also how have they changed the pH during simulation. Are there any experimental data / results to support these pH values?
4. Fig 3 (A) and 4 (A) – rmsd of same protein is different or they represent different simulations / protein structure? Kindly explain.
5. How are the resultant compounds better than ones shown in Garcia *et al.*, (2019) ^1^ and Carter *et al.,* (2021) ^2^?

Minor points: authors can check Kumari *et al., *(2023) ^4^ and Saha & Nath Jha (2023) ^4^, for workflow and *Leishmania *related updates.

Is the work clearly and accurately presented and does it cite the current literature?

Partly

If applicable, is the statistical analysis and its interpretation appropriate?

Not applicable

Are all the source data underlying the results available to ensure full reproducibility?

Partly

Is the study design appropriate and is the work technically sound?

Partly

Are the conclusions drawn adequately supported by the results?

Partly

Are sufficient details of methods and analysis provided to allow replication by others?

No

Reviewer Expertise:

Computational Biophysics, Molecular Dynamics simulations, Drug design and discovery

I confirm that I have read this submission and believe that I have an appropriate level of expertise to confirm that it is of an acceptable scientific standard, however I have significant reservations, as outlined above.

## References

[1] : Leishmania infantum arginase: biochemical characterization and inhibition by naturally occurring phenolic substances. *J Enzyme Inhib Med Chem* .2019;34(1) : 10.1080/14756366.2019.1616182 1100-1109 10.1080/14756366.2019.1616182 31124384PMC6534257

[2] : Natural Products That Target the Arginase in Leishmania Parasites Hold Therapeutic Promise. *Microorganisms* .2021;9(2) : 10.3390/microorganisms9020267 10.3390/microorganisms9020267 33525448PMC7911663

[3] : Leishmaniasis: Omics Approaches to Understand its Biology from Molecule to Cell Level. *Curr Protein Pept Sci* .2023;24(3) : 10.2174/1389203724666230210123147 229-239 10.2174/1389203724666230210123147 36809951

[4] : Computational multi-target approach to target essential enzymes of Leishmania donovani using comparative molecular dynamic simulations and MMPBSA analysis. *Phytochem Anal* .2023; 10.1002/pca.3213 10.1002/pca.3213 36760044

# Response

**Published:** 2023-07-10

**Authors:** Miguel Angel Chavez-Fumagalli

**Affiliations:** Universidad Catolica de Santa Maria, Peru

1)  Authors have tried drug repurposing for Leishmaniasis and targeted arginase of L. infantum. There are some questions to be answered by authors:

**ANSWER: I appreciate your evaluation and helpful criticism. To enhance the manuscript, we have taken into consideration your suggestions.**

 2) Which pdb structure (pdb id) of human L-arginine was selected https://www.uniprot.org/uniprotkb/P05089/entry#structure) for docking?

**ANSWER: I appreciate your analysis. As mentioned in the methods section, we obtained the Homo sapiens ARG's FASTA sequence, carried out automated modeling** **in Swiss-Model, and chose the top models.**

 3) What is the sequence & structure level similarity between human and L. infantum ARG proteins? Give the justification of selection for ARG protein as target for drug discovery based on that similarity.

**ANSWER: I value what you pointed out. We addressed this crucial issue raised by the reviewer in the text's results sections by adding comments. While the discussion and introduction sections of the work have advocated for the choice of ARG as a target.**

 4) Any reason for selection of two pH values (2 & 7) and also how have they changed the pH during simulation. Are there any experimental data / results to support these pH values?

**ANSWER: I value what you pointed out. Our research focused on a significant obstacle to the development of leishmaniasis drugs: the requirement that drugs work in the acidic environment of the phagolysosome. This important information is included in the introduction and discussion portion of the article. The pH ranges selected were intended to compare variations in the target dynamics and complex stabilities under the two settings.**

 5) Fig 3 (A) and 4 (A) – rmsd of same protein is different or they represent different simulations / protein structure? Kindly explain.

**ANSWER: I appreciate your perceptive observation. Figure 4 contrasts the target dynamics and complex stabilities at pH 2 or 7 (with the two molecules and without), lasting within nanoseconds. Figure 3 depicts the target at both pHs without binding a molecule.**

 6) How are the resultant compounds better than ones shown in Garcia et al., (2019)1 and Carter et al., (2021)2?

**ANSWER: I appreciate your astute observation. Compared to the results obtained for most of the chemicals mentioned in the cited studies, our findings are, nonetheless, preliminary. Since 2000, numerous in vitro, in vivo, and silico studies have been conducted to assess natural product’s potential as an antileishmanial substance. as a single module, using nanocarriers and delivery systems [Alanazi AD, Ben Said M. Plant Bioactive Ingredients in Delivery Systems and Nanocarriers for the Treatment of Leishmaniasis: An Evidence-Based Review. Iran J Parasitol. 2022 Oct-Dec;17(4):458-472. doi: 10.18502/ijpa.v17i4.11272. PMID: 36694570; PMCID: PMC9825702.]. The approach outlined in this study aims to use a logical search for novel candidate molecules in which structural similarities to compounds that have been extensively investigated may result in novel molecular activities. While choosing substances that showed the potential for oral absorption and were selective against the parasite target. Malvidin performed the best in this case, however, it should first be studied in vitro and in vivo in order to meet the criteria for a candidate.**

 7) Minor points: authors can check Kumari et al., (2023)4 and Saha & Nath Jha (2023)4, for workflow and Leishmania related updates.

**ANSWER: We value your highly regarded suggestion. I will definitely consider it in future studies.**

# Peer review: Reviewer response for version 2

**DOI:** 10.5256/f1000research.148092.r175026

**Published:** 2023-05-31

**Authors:** Francisco Centeno

**Affiliations:** 1 Departamento de Bioquímica y Biología Molecular y Genética, Facultad de Ciencias, Universidad de Extremadura, Badajoz, Extremadura, Spain

I have no further comments to make.

Is the work clearly and accurately presented and does it cite the current literature?

Yes

If applicable, is the statistical analysis and its interpretation appropriate?

Not applicable

Are all the source data underlying the results available to ensure full reproducibility?

Partly

Is the study design appropriate and is the work technically sound?

Yes

Are the conclusions drawn adequately supported by the results?

Partly

Are sufficient details of methods and analysis provided to allow replication by others?

Yes

Reviewer Expertise:

Bioinformatics, Arginase, Protein structure

I confirm that I have read this submission and believe that I have an appropriate level of expertise to confirm that it is of an acceptable scientific standard.

# Peer review: Reviewer response for version 1

**DOI:** 10.5256/f1000research.142667.r165815

**Published:** 2023-04-11

**Authors:** Francisco Centeno

**Affiliations:** 1 Departamento de Bioquímica y Biología Molecular y Genética, Facultad de Ciencias, Universidad de Extremadura, Badajoz, Extremadura, Spain

The authors of this study employed various CADD techniques to screen structural analogs of natural products that could potentially serve as new therapeutic agents against leishmaniasis. The study is well-oriented and well-planned, particularly about how the screening of candidate molecules has been carried out from various compound libraries. This last point seems to be the best and most consistent aspect of the work in its current format.

 However, all the work on simulating the molecular dynamics of the interaction between the ligands and the arginase of *L. infantum* is based on the structure determined in Swiss-Model, using the structure of the arginase of *L. mexicana *as a template. Since the entire study relies on this structure determination being accurate, it is crucial that sufficient structural data be incorporated into this work to convincingly demonstrate that the structure determination obtained in silico is the best possible.

 In this regard, some issues also arise that should be addressed:

1. Have any refinements been made to the structure initially obtained from Swiss-Model to improve it?
2. Why has only the arginase from *L. infantum* been chosen for dynamic simulation studies, and not the arginases from *L. brasiliensis* and/or *L. mexicana*, whose structures are known? It would be interesting to compare the dynamic data obtained for *L. infantum* with those obtained for *L. mexicana*, whose structure is known.

Another important point that limits the work presented, in which I agree with the authors, is that the time scales obtained for the stabilities of the arginase-ligand complexes are surprisingly short. Although the authors discussed this in the discussion section, their explanation seems implausible to me. To clarify this point, I suggest comparing the stability times of the complexes with those obtained with the physiological ligand, arginine, and comparing the arginine complexes with human and *L. mexicana* arginases, both of which are proteins with known structures.

Is the work clearly and accurately presented and does it cite the current literature?

Yes

If applicable, is the statistical analysis and its interpretation appropriate?

Not applicable

Are all the source data underlying the results available to ensure full reproducibility?

Partly

Is the study design appropriate and is the work technically sound?

Yes

Are the conclusions drawn adequately supported by the results?

Partly

Are sufficient details of methods and analysis provided to allow replication by others?

Yes

Reviewer Expertise:

Bioinformatics, Arginase, Protein structure

I confirm that I have read this submission and believe that I have an appropriate level of expertise to confirm that it is of an acceptable scientific standard, however I have significant reservations, as outlined above.

# Response

**Published:** 2023-05-11

**Authors:** Miguel Angel Chavez-Fumagalli

**Affiliations:** Universidad Catolica de Santa Maria, Peru

**REVIEWER 1**

 The authors of this study employed various CADD techniques to screen structural analogs of natural products that could potentially serve as new therapeutic agents against leishmaniasis. The study is well-oriented and well-planned, particularly about how the screening of candidate molecules has been carried out from various compound libraries. This last point seems to be the best and most consistent aspect of the work in its current format.

**ANSWER: Thank you for your assessment and constructive criticism. We have included all the recommendations described below to improve the manuscript.**

 However, all the work on simulating the molecular dynamics of the interaction between the ligands and the arginase of L. infantum is based on the structure determined in Swiss-Model, using the structure of the arginase of L. mexicana as a template. Since the entire study relies on this structure determination being accurate, it is crucial that sufficient structural data be incorporated into this work to convincingly demonstrate that the structure determination obtained in silico is the best possible.

**ANSWER: I appreciate your observation. We made comments in the methods sections of the text to address this crucial point brought up by the reviewer.**

 In this regard, some issues also arise that should be addressed:

 Have any refinements been made to the structure initially obtained from Swiss-Model to improve it?

**ANSWER: Thanks for your assessment. As stated in the methods section we performed an automated modeling analysis and selected the best models for each target.**

 Why has only the arginase from L. infantum been chosen for dynamic simulation studies, and not the arginases from L. brasiliensis and/or L. mexicana, whose structures are known? It would be interesting to compare the dynamic data obtained for L. infantum with those obtained for L. mexicana, whose structure is known.

**ANSWER:** **Thank you for your keen observation. We chose the ARG from L. infatum since it is the species that causes leishmaniasis, it has the most severe form of the disease, and if VL is confirmed, therapy against it is required. While many of the natural compounds included in this study bind to highly conserved residues in Leishmania ARG, according to docking experiments conducted by numerous labs. In fact, a lot of them seem to use the same residues regardless of their structural classification [da Silva, Edson Roberto, et al. "Cinnamic acids derived compounds with antileishmanial activity target Leishmania amazonensis arginase." Chemical Biology & Drug Design 93.2 (2019): 139-146.]. Additionally, we restricted our research to New World Leishmaniasis because, as far as we are aware, no other crystal structures outside L. mexicana ARG have been deposited.**

 Another important point that limits the work presented, in which I agree with the authors, is that the time scales obtained for the stabilities of the arginase-ligand complexes are surprisingly short. Although the authors discussed this in the discussion section, their explanation seems implausible to me. To clarify this point, I suggest comparing the stability times of the complexes with those obtained with the physiological ligand, arginine, and comparing the arginine complexes with human and L. mexicana arginases, both of which are proteins with known structures.

**ANSWER: I appreciate your observation. We made comments in the methods and results sections of the text to address this crucial point brought up by the reviewer. We carried out the MDS at 100 ns under the same conditions as those mentioned earlier in the manuscript. Since the parasite replicates in the macrophages, stability at low pH is the requirement that a medication must meet, as stated in the article. Several of the natural products that were employed as models for the selection of the structural analogs have demonstrated intracellular action in vitro.**
